# Supplementary material for: Causal Relationship between Aging and Anorexia Nervosa: A White-Matter-Microstructure-Mediated Mendelian Randomization Analysis
Source: Biomedicines. 2024 Aug 16;12(8):1874. doi: 10.3390/biomedicines12081874 (PMC11351342; doi:10.3390/biomedicines12081874)
Supplement: Supplementary file 1 [file biomedicines-12-01874-s001.zip › Supplementary Figure/Figure S9/Leaveoneout plot of MR analysis for ubm-b-1685 to AN.pdf]

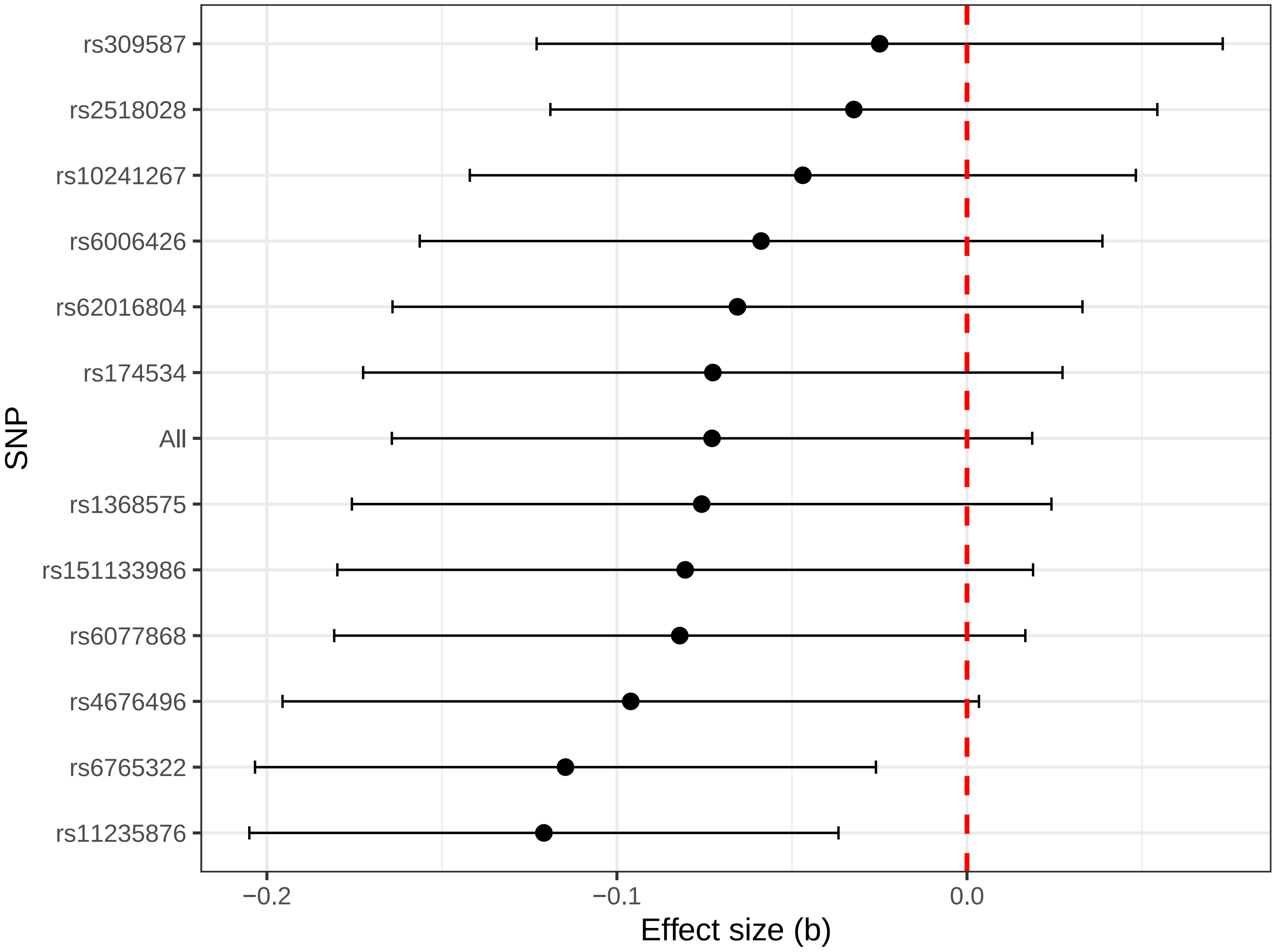
rs309587
rs2518028
rs10241267
rs6006426
rs62016804
rs174534
All
rs1368575
rs151133986
rs6077868
rs4676496
rs6765322
rs11235876
SNP
−0.2
−0.1
0.0
Effect size (b)
